# Antispasmodic Effects and Action Mechanism of Essential Oil of *Chrysactinia mexicana* A. Gray on Rabbit Ileum

**DOI:** 10.3390/molecules21060783

**Published:** 2016-06-16

**Authors:** Daniel Zavala-Mendoza, Laura Grasa, Miguel Ángel Zavala-Sánchez, Salud Pérez-Gutiérrez, María Divina Murillo

**Affiliations:** 1 Departamento de Farmacología y Fisiología (Fisiología), Facultad de Veterinaria, Universidad de Zaragoza, Miguel Servet 177, 50013 Zaragoza, Spain; menzd_che@yahoo.com.mx (D.Z.-M.); divinamurillo@hotmail.com (M.D.M.); 2 Dirección General de Servicios Educativos Iztapalapa, Av. Rojo Gómez No. 1149, Col. Barrio San Pedro, Del Iztapalapa 09300 México, DF, Mexico; 3 Instituto de Investigación Sanitaria de Aragón (IIS-Aragón), 50013 Zaragoza, Spain; 4 Instituto Agroalimentario de Aragón (IA2), 50013 Zaragoza, Spain; 5 Departamento de Sistemas Biológicos, Universidad Autónoma Metropolitana-Xochimilco, Calzada del Hueso 1100, Colonia Villa Quietud, Coyoacán, 09340 Mexico, DF, Mexico; migzavalasan@gmail.com (M.Á.Z.-S.); msperez@correo.xoc.uam.mx (S.P.-G.)

**Keywords:** *Chrysactinia mexicana*, essential oil, antispasmodic effect, ileum, rabbit, intestinal motility

## Abstract

The *Chrysactinia mexicana* A. Gray *(C. mexicana)* plant is used in folk medicine to treat fever and rheumatism; it is used as a diuretic, antispasmodic; and it is used for its aphrodisiac properties. This study investigates the effects of the essential oil of *C. mexicana* (EOCM) on the contractility of rabbit ileum and the mechanisms of action involved. Muscle contractility studies *in vitro* in an organ bath to evaluate the response to EOCM were performed in the rabbit ileum. EOCM (1–100 µg·mL^−1^) reduced the amplitude and area under the curve of spontaneous contractions of the ileum. The contractions induced by carbachol 1 µM, potassium chloride (KCl) 60 mM or Bay K8644 1 µM were reduced by EOCM (30 µg·mL^−1^). Apamin 1 µM and charybdotoxin 0.01 µM decreased the inhibition induced by EOCM. The d-cAMP 1 µM decreased the inhibition induced by EOCM. l-NNA 10 µM, Rp-8-Br-PET-cGMPS 1 µM, d,l-propargylglycine 2 mM, or aminooxyacetic acid hemihydrochloride 2 mM did not modify the EOCM effect. In conclusion, EOCM induces an antispasmodic effect and could be used in the treatment of intestinal spasms or diarrhea processes. This effect would be mediated by Ca^2+^, Ca^2+^-activated K^+^ channels and cAMP.

## 1. Introduction

*Chrysactinia mexicana* A. Gray *(Asteraceae)* is a plant distributed throughout central and northern Mexico [1]. This plant is used to treat respiratory diseases, skin infections, fever and rheumatism [2]. Furthermore, it has diuretic, antispasmodic and aphrodisiac properties, and it has been used as a general tonic, an energetic or as a stimulating agent [3]. Several antifungal functions or activities of *C. mexicana* essential oil, isolated by Cardenas and coworkers, have also been reported [4]. Although the toxicity of the essential oil of *C. mexicana* has not been reported, an aqueous extract of *C. mexicana* has shown no toxicity in mice. Mice treated with the aqueous extract of *C. mexicana* by gavage (dose range 1–200 mg·mL^−1^) showed no signs of neurotoxicity, such as convulsion, stereotyped behavior or ataxia. The LD_50_ of the extract was estimated to be greater than 5000 mg·Kg^−1^. In addition, this extract did not produce damage in the hepatic functions or in the morphology of the liver and the kidney [3]. Previous studies have shown that among the chemical constituents of *C. mexicana* are flavonoid glycosides [5], thiophenes [6] and terpenes [7]. More recently, eucalyptol, piperitone, and linalyl acetate were found to be major components of essential oil of *C. mexicana* [4,8]. Guerra-Boone and coworkers showed that sylvestrene and limonene are main components of the essential oil of *C. Mexicana* [9]. The LD_50_ of eucalyptol, one of the major components of the EOCM, is estimated as 3849 mg·kg^−1^. Subacute toxicity studies showed that the dose of 21.38 mg·kg^−1^ per day of eucalyptol causes no damage on mouse liver and kidneys [10].

The rabbit small intestine represents a good model to study the intestinal motility. First, the intestinal motility of rabbit exhibits cyclic, phasic and rhythmic spontaneous contractions that are highly reproducible. Second, this model is widely used to study different substances or new drugs. Finally, our group has characterized these spontaneous contractions and we have studied the mechanisms of action involved in them. The spontaneous motility and contractility of rabbit intestine is mediated by the influx of extracellular Ca^2+^, intracellular Ca^2+^ release [11] and K^+^ channels [12]. Different agents induce relaxing responses in the rabbit intestine as sodium nitroprusside (nitric oxide donor), trolox (analogue of vitamin E) or quercetin and genistein (flavonoids) [13,14,15]. 

Folk medicine has been using *C. mexicana* for the treatment of different diseases and symptoms, but many of these effects have not been scientifically investigated. In this present work, we studied the effect of EOCM on the longitudinal smooth muscle of rabbit ileum and its possible mechanisms of action.

## 2. Results

### 2.1. Effect of EOCM on Ileum Motility

**Figure 1:**
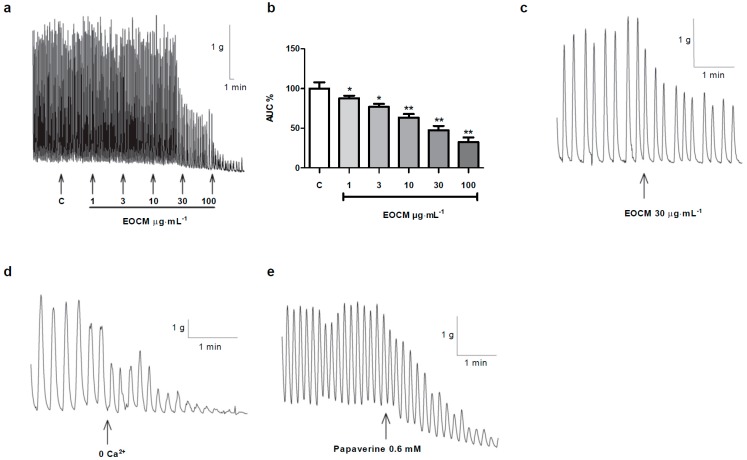
Effect of EOCM, Ringer Krebs free-Ca^2+^ and 1 mM EGTA, and papaverine on longitudinal smooth muscle of rabbit ileum. (**a**) Recordings of cumulative concentration-response curve of EOCM (1–100 µM); (**b**) Histograms showing the values of cumulative concentration-response curve of EOCM (1–100 µM). The columns show the mean values, and vertical bars indicate SEM of 14 segments; (**c**) Recording of EOCM 30 µg·mL^−1^; (**d**) Recording of Ringer Krebs free-Ca^2+^ and 1 mM EGTA (0 Ca^2+^); (**e**) Recording of papaverine 0.6 mM. Arrowheads indicate the addition of agents. * *p* < 0.05; ** *p* < 0.01 *vs.* control.

We studied the effect of EOCM on the spontaneous motility of rabbit ileum. EOCM (1–100 µg·mL^−1^) reduced the area under the curve (AUC) (Figure 1a, b) and the amplitude but not the frequency of spontaneous contractions of longitudinal smooth muscle of the rabbit ileum (Table 1 and Figure 1a). This effect was concentration-dependent and reversible after washing. As EOCM was dissolved in DMSO, we studied the effect per se of DMSO on the spontaneous contractions of the ileum. The AUC, amplitude and the frequency of spontaneous contractions on longitudinal smooth muscle were not modified in the presence of DMSO (Table 2).

### 2.2. Comparison between the Effects of Free-Calcium Krebs, Papaverine and EOCM on Smooth Muscle of Ileum

We compared the effect of EOCM with the effects of free-Ca^2+^ Krebs and papaverine on spontaneous motility. In free-Ca^2+^ Krebs with EGTA 1 mM, the amplitude, frequency and the AUC of spontaneous contractions were reduced, with the inhibition of the AUC approximately 70% (29.2% ± 5.9%, *n* = 23) (Figure 1d). Papaverine 0.6 mM (a smooth muscle relaxant) reduced the amplitude, frequency, tone and AUC (38.2% ± 4.1%, *n* = 25) of spontaneous contractions of the ileum (Figure 1e). EOCM 30 µg·mL^−1^, as mentioned above, decreased the spontaneous contractions, thus evoking a relaxing response (Figure 1c). The shape of the response on the smooth muscle of the ileum to the three agents was different, with the inhibition evoked by EOCM, which is lower than free-Ca^2+^ Krebs with EGTA or papaverine. 

### 2.3. Effect of EOCM on Carbachol- or KCl-Induced Contractions

**Figure 2:**
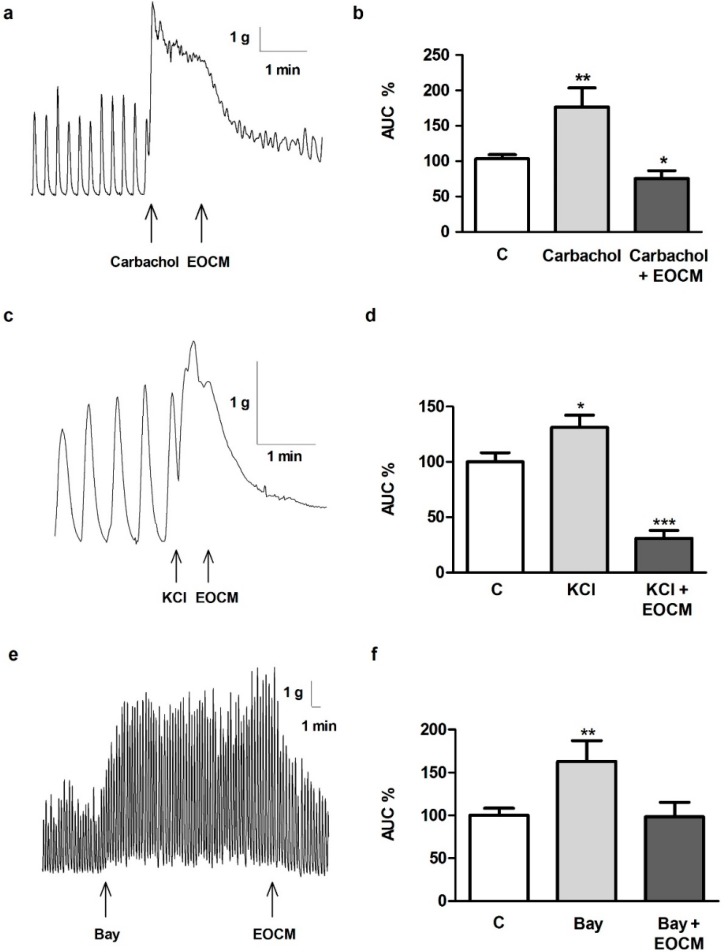
Effect of carbachol 1 µM, KCl 60 mM, Bay K8644 1 µM before the addition of EOCM 30 µg·mL^−1^ on longitudinal smooth muscle of rabbit ileum. (**a**,**c**,**e**) Recordings of carbachol, KCl and Bay K8644 before the addition of EOCM; (**b**,**d**,**f**) Histograms showing the values of control (**c**), carbachol, and carbachol + EOCM; KCl, and KCl + EOCM; and Bay K8644, and Bay + EOCM, respectively. The columns show the mean values, and vertical bars indicate SEM of 14 segments. Arrowheads indicate the addition of agents. * *p* < 0.05; ** *p* < 0.01; *** *p* < 0.001 *vs.* control.

To examine the effect of EOCM on contractile agonists, segments of ileum were pre-incubated with EOCM before the addition of carbachol or KCl. The contractile responses induced by carbachol 1 µM or KCl 60 mM were reduced after the addition of EOCM (30 µg·mL^−1^) on the longitudinal muscle of the ileum (Figure 2a–d). Furthermore, the previous incubation of EOCM for 15 min decreased the contractions induced by carbachol 1 µM (75.7% ± 7.3%, *n* = 16, *p* < 0.05 *vs.* control) or KCl 60 mM (21.9% ± 5.1%, *n* = 9, *p* < 0.001 *vs.* control).

### 2.4. Role of Ca^2+^ on the Effect of EOCM

The role of Ca^2+^ on the EOCM relaxant effect was tested on segments of ileum, which were incubated with different agonists and antagonists of calcium. The incubation of ileal segments with Bay K8644 1 µM (an activator of Ca^2+^) for 15 min increased the AUC of spontaneous contractions of the ileum. The addition of EOCM (30 µg·mL^−1^) after Bay K8644 restored the control values (Figure 2e,f). The addition of verapamil 0.1 µM (an antagonist of voltage-dependent Ca^2+^ channels) after Bay K8644 also restored the control values (101.7% ± 12.3%, *n* = 6). Trifluoperazine 1 µM (a calmodulin antagonist) did not modify the EOCM effect on the AUC of the spontaneous contractions of the ileum (trifluoperazine 87.4% ± 5.6%, EOCM 56.0% ± 7.5% *vs.* trifluoperazine + EOCM 52.9% ± 7.2%, *n* = 6).

### 2.5. Role of K^+^ Channels on the Effect of EOCM

**Figure 3:**
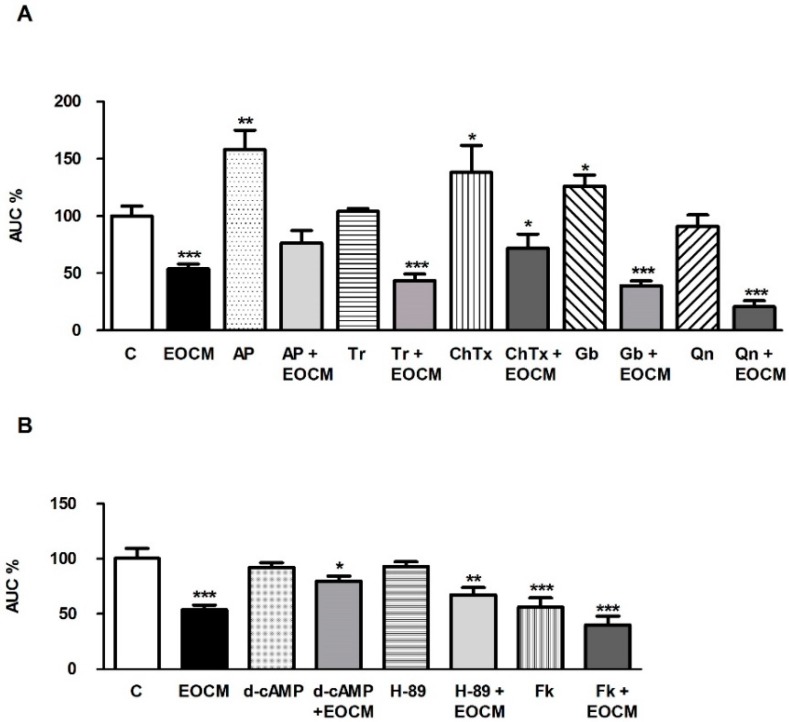
(**A**) Histograms showing the effect of K^+^ channel inhibitors apamin (Ap), Tram-34, charybdotoxin (ChTx), glibenclamide (Gb) and quinine (Qn) on the inhibition induced by EOCM 30 µg·mL^−1^ on the longitudinal smooth muscle of rabbit ileum; (**B**) Histograms of the effect of cAMP-related agents d-cAMP, H-89 and forskolin (Fk) on the inhibition induced by EOCM 30 µg·mL^−1^ on the longitudinal smooth muscle of rabbit ileum. The columns show the mean values, and vertical bars indicate SEM of 14 segments. * *p* < 0.05; ** *p* < 0.01, *** *p* < 0.001 *vs.* control.

In order to study the role of K^+^ channels on the EOCM relaxant effect, segments of ileum were incubated with different inhibitors of K^+^ channels. The effect of K^+^ channel inhibitors on spontaneous contractions and EOCM (30 µg·mL^−1^) response in the ileum is shown in Figure 3A. Apamin 1 µM (Ap, a potent and selective inhibitor of small-conductance Ca^2+^-activated K^+^ channels) increased the AUC of the spontaneous contractions of ileum, indicating a per se effect of this K^+^ channel inhibitor on spontaneous contractions (Figure 3A). However, Tram-34 1 µM (Tr, a selective and potent inhibitor of intermediate-conductance Ca^2+^-activated K^+^ channels), charybdotoxin 0.01 µM (ChTx, a specific inhibitor of intermediate- and large-conductance Ca^2+^-activated K^+^ channels), glibenclamide 0.1 µM (Gb, an inhibitor of ATP-dependent K^+^ channels) and quinine 10 µM (Qn, an inhibitor of voltage-sensitive K^+^ channels) did not modify the AUC on spontaneous contractions of ileum. Only apamin and charybdotoxin reduced it slightly, but they significantly reduced the EOCM effect on the AUC of the ileum spontaneous contractions (Figure 3A).

### 2.6. Role of cAMP on the Effect of EOCM

We investigated the involvement of cAMP on the EOCM relaxant response in the rabbit ileum. The addition of d-cAMP 1 µM (a cAMP analog) or H-89 1 µM (a potent inhibitor of cAMP-dependent protein kinase) did not modify the AUC of the ileal spontaneous contractions. The previous incubation with d-cAMP for 15 min decreased the EOCM (30 µg·mL^−1^) and induced inhibition on the AUC of spontaneous contractions of the ileum (Figure 3B).

Forskolin 10 µM (an activator of adenil cyclase) reduced the AUC of spontaneous contractions of the ileum. The previous incubation of the ileum with forskolin did not modify the effect of EOCM (30 µg·mL^−1^) on the AUC of spontaneous contractions of the ileum (Figure 3B).

### 2.7. Role of Nitric Oxide and cGMP on the Effect of EOCM

**Figure 4:**
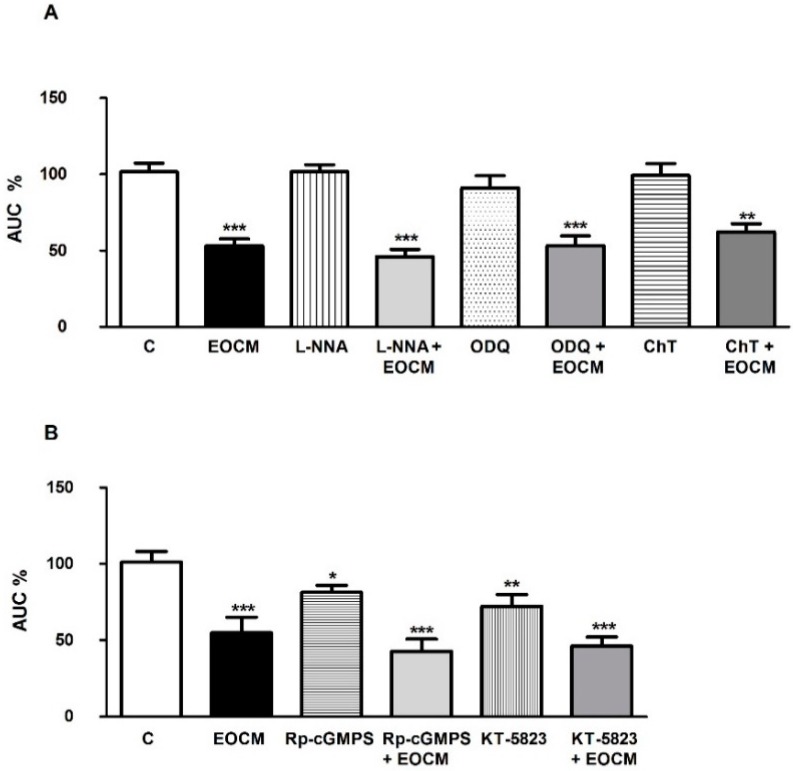
(**A**) Histograms showing the effects of l-NNA, ODQ, and chelerythrine on the inhibition induced by EOCM 30 µg·mL^−1^ on the longitudinal smooth muscle of rabbit ileum; (**B**) Histograms of the effects of Rp-8-Br-PET-cGMPS and KT-5823 on the inhibition induced by EOCM 30 µg·mL^−1^ on the longitudinal smooth muscle of rabbit ileum. The columns show the mean values, and vertical bars indicate SEM of eight segments. * *p* < 0.05; ** *p* < 0.01, *** *p* < 0.001 *vs.* control.

We investigated the involvement of nitric oxide and cGMP on the EOCM relaxant response in the rabbit ileum. The AUC of spontaneous contractions on the longitudinal muscle of the ileum was not modified by l-NNA 10 µM (a NOS inhibitor), ODQ 1 µM (a potent and selective inhibitor of guanylyl cyclase) and chelerythrine 1 µM (a protein kinase C inhibitor) (Figure 4A), but was diminished by Rp-8-Br-PET-cGMPS 1 µM (a cGMP analog) and KT-5823 1 µM (a selective inhibitor of protein kinase G) (Figure 4B). The incubation of ileum segments for 15 min with l-NNA, ODQ, chelerythrine, Rp-8-Br-PET-cGMP or KT-5823 did not modify the EOCM effect on the AUC of spontaneous contractions (Figure 4A,B).

### 2.8. Role of other Agents on the Effect of EOCM

Finally, we examined whether myosin light chain phosphatase, Rho-kinase or hydrogen sulphide participated in the EOCM relaxant effect in the rabbit ileum. Okadaic acid 0.1 µM (Ok, a potent inhibitor of myosin light chain phosphatase) did not alter the AUC of spontaneous contractions of the ileum in the absence (control 100 *vs.* okadaic acid 100.9% ± 2.8%, *n* = 7) or the presence of EOAM (EOCM 59.2% ± 8.6% *vs.* okadaic acid + EOCM 56.9% ± 9.2%, *n* = 7). Y-27632 1 µM (an inhibitor of Rho-kinase) reduced the AUC of spontaneous contractions of the ileum (control 100%, Y-27632 80.2% ± 4.6% with *p* < 0.05 *vs.* control, *n* = 7). Y-27632 1 µM did not modify the inhibition induced by EOCM (30 µg·mL^−1^) (EOCM 56.7% ± 5.2%, Y-27632+EOCM 47.1% ± 6.1% *p* < 0.001 *vs.* control, *n* = 7).

d,l-propargylglycine 2 mM (PAG, an irreversible inhibitor of the enzyme cystathionine γ-lyase), aminooxyacetic acid hemihydrochloride 2 mM (AOAA, an inhibitor of cystathionine β-synthase), PGA 2 mM+ AOAA 2 mM, tetrodotoxin (TTX) 1 µM + l-NNA 10 µM+PGA 2 mM, TTX 1 µM + l-NNA 10 µM + AOAA 2 mM or TTX 1 µM + l-NNA 10 µM + PGA 2 mM + AOAA 2 mM did not alter the AUC of the spontaneous contractions of the ileum (control 100% *vs.* PAG 106.0% ± 5.4%, AOAA 94.5% ± 3.8%, PAG + AOAA 89.6% ± 3.3%, TTX + l-NNA + PGA 97.5% ± 4.1%, TTX + l-NNA + AOAA 94.8% ± 7.0%, TTX + l-NNA + PGA + AOAA 87.7% ± 10.6%; *n* = 8). Furthermore, ileal segments incubated for 15 min with PAG, AOAA, PAG + AOAA, TTX + l-NNA + PGA, TTX + l-NNA + AOAA or TTX + l-NNA + PGA + AOAA did not modify the EOCM (30 µg·mL^−1^)-evoked inhibition on the AUC of the ileal spontaneous contractions (EOCM 51.1% ± 4.9% *vs.* PAG + EOCM 50.1% ± 7.3%, AOAA + EOCM 43.7% ± 4.6%, PGA + AOAA + EOCM 42.6% ± 3.3%, TTX + l-NNA + PGA + EOCM 43.1% ± 4.3%, TTX + l-NNA + AOAA + EOCM 45.1% ± 4.8%, TTX + l-NNA + PGA + AOAA + EOCM 31.0% ± 4.4% *p* < 0.001, *n* ≥ 7).

## 3. Discussion

*Chrysactinia* *mexicana* is a plant used in folk medicine with antispasmodic actions. EOCM induced relaxation on the longitudinal smooth muscle of the rabbit ileum. The relaxations of intestinal muscle are caused by inhibition of the influx or release of Ca^2+^ to cytosol [11]. Furthermore, cAMP participates in the relaxations of the smooth muscle evoked by different agents. The relaxing response of the rabbit’s ileal smooth muscle agrees with the antispasmodic function attributed to *C. mexicana* as a medicinal plant, although this effect has not been previously tested. The EOCM-induced relaxation on the smooth muscle of the ileum was lower than the relaxation evoked in free-Ca^2+^ Krebs with EGTA. The EOCM relaxation was similar in shape to the papaverine response on the ileal smooth muscle, although papaverine evoked a decrease in muscle tone (Figure 1). Papaverine is a relaxant agent of smooth muscle and it is used as a positive control for comparing relaxing responses. The relaxing response obtained in this study agrees with the relaxing effect observed with the extract of *Artemisia ludoviciana*, *Artemisia* *maritima* and *Ferula heuffelii* in the intestine [16,17,18].

Contractile agents such as carbachol or KCl induced contractions or spasms in intestinal smooth muscle. In this study, the carbachol- or KCl-induced contractions were decreased by EOCM. Furthermore, the increase of spontaneous contractions in the rabbit ileum induced by Bay K8644 was restored to control values by EOCM or verapamil but the contractions were not modified by trifluoperazine. This finding concurs with the essential oil of *Pistacia integerrima,* which induces the inhibition of the transient contraction of acetylcholine and the relaxation of the Bay K8644-precontracted isolated guinea pig ileum, and these authors suggest that this effect is related to its ability to inhibit the l-subtype Ca^2+^ channels [19]. Furthermore, the extracts of *Artemisia maritima* and *Artemisia ludoviciana* induce relaxing effects on the contraction produced by potassium chloride in rabbit jejunum and rat ileum [16,17]. Our results suggest that the effect induced by EOCM on spontaneous contractions and on carbachol- or K^+^-induced contractions are antispasmodic or relaxing responses in the longitudinal smooth muscle of the rabbit ileum. Moreover, EOCM and verapamil reduced the increase of the AUC on the smooth muscle of the ileum evoked by the analog of Ca^2+^, Bay K8644, and in these effects the Ca^2+^ enter is involved.

In this study, apamin and charybdotoxin partly decreased but tram-34, glibenclamide or quinine did not alter the effect of EOCM on the smooth muscle of the ileum. Similar results were obtained with glibenclamide or TEA (a non-selective inhibitor of K^+^ channels), agents that did not inhibit the spasmolytic activity of *Artemisia copa* [20] or *Mentha pulegium* [17]. Also, dichloromethanic extract of *Artemisia ludoviciana spp. mexicana* inhibits the rat ileum spontaneous contraction and this inhibition was not modified by ODQ, indometacine, l-NAME, glibenclamide and 2-aminopyridine [17].

On the other hand, dibutyryl-cAMP and H-89 reduced but forskolin increased the inhibition induced by EOCM. Furthermore, Rp-8-Br-PET-cGMPS, ODQ, chelerythrine, KT-5823, or l-NNA did not modify the EOCM-induced effect on spontaneous contractions in the ileum. Similar results were obtained with l-NAME or ODQ which did not modify the inhibitory effect evoked by *Achillea millefolium* and *Artemisia ludoviciana* in the rat ileum [17,21]. We propose that the EOCM effect is mediated in part by cAMP, but not cGMP or NO.

Contractile stimuli can sensitize myosin to Ca^2+^ by activating RhoA/Rho-kinase which inhibits myosin light chain phosphatase (MLCP) activity. Conversely, smooth muscle relaxation results from a decrease in the concentration of cytosolic Ca^2+^ and/or a reduced Ca^2+^ sensitivity of the contractile apparatus.

In this way, we examined other possible mechanisms in the EOCM effect. Okadaic acid (inhibitor of MLCP) did not alter the spontaneous contractions of the rabbit ileum and the EOCM effect. Y-27632 (inhibitor of Rho-kinase) inhibited the spontaneous contractions of the rabbit ileum, but it did not modify the EOCM effect. Similar results were obtained in inhibiting Rho-kinase or MCLP and it had no effect on the nitric oxide–induced relaxation in the circular smooth muscle of the rat colon [22]. 

PAG (inhibitor of the enzyme cystathionine γ-lyase), AOAA (inhibitor of cystathionine β-synthase) or PAG + AOAA, did not alter the spontaneous contractions of the rabbit ileum and the EOCM effect. These agents inhibit the synthesis of hydrogen sulfide. Hydrogen sulfide has been suggested as a gaseous neuromodulator in mammals and evokes the relaxations of smooth muscle [23].

Previously, it has been described that the extract of the aerial part of *C. mexicana* has different components such as flavonoid glycosides [5], thiophenes [6], terpenes [7]. Furthermore, the chemical composition of the essential oil of *C. mexicana* isolated in this work was analyzed by gas chromatography–mass spectrometry; the components were eucalyptol (28.7%), α-terpeniol (9.9%) and piperitone (52.1%) [8]. Some components of *C. mexicana* have been described in other plants. The effects of the essential oil of *Eucalyptus tereticornis*, with the main constituent 1,8-cineole or eucalyptol, enhanced and reduced acetylcholine-induced contractions dependent on the concentration, at lower and higher concentrations respectively, on guinea pig tracheal smooth muscle [24]. The addition of 1,8-cineole relaxes the tracheal rings’ carbachol-precontracted or K^+^ contractions [25]. Pure commercial piperitone, eucalyptol, and alpha-terpineol, which are the major constituents of *Casimiroa pringlei* essential oil, cause an antispasmodic effect on the rat uterus [26]. Flavonoids are phenolic compounds with effects on intestinal peristalsis [27]. Flavonoids such as genistein and quercetin reduced the amplitude of spontaneous contractions in the smooth muscle of rabbit duodenum, but they did not modify the frequency, these effects being similar to those found for EOCM. The effect of genistein is mediated by Ca^2+^ and K^+^ channels, while the effect of quercetin is mediated by cAMP and protein kinase A [15]. Trolox decreases the amplitude and frequency of spontaneous contractions and the acetylcholine-induced contractions [14]. However, sodium nitroprusside reduces the amplitude, frequency and tone of spontaneous contractions [13]. The EOCM-induced relaxation may be due to components such as piperitone, eucalyptol and alpha-terpineol, which are described as relaxing agents.

In conclusion, the antispasmodic effect induced by EOCM involves Ca^2+^, Ca^2+^-activated K^+^ channels and cAMP and might be used in the treatment of intestinal spasms or diarrhea processes.

## 4. Materials and Methods

### 4.1. Plant Material

*C. mexicana* leaves were collected in July 2012, in Guadalcázar, state of San Luis Potosí (Mexico). The material was authenticated by taxonomist José García-Pérez and a voucher specimen (SLPM37571) was deposited at the Isidro Palacios Herbarium of the Universidad Autónoma de San Luis Potosí. Essential oil of *Chrysactinia mexicana* Gray (EOCM) was isolated and labeled in Laboratory N008 (Organic Chemistry Investigation and Natural Products), Department of Biological Systems of Universidad Autónoma Metropolitana Unidad Xochimilco (Mexico) [8].

### 4.2. Preparation of EOCM for the Test

Dimethyl sulfoxide (DMSO) was used to solve the EOCM. Cumulative concentration-response curves to DMSO were also performed to test the per se effect of DMSO.

### 4.3. Animals

All procedures were carried out under Project License PI03/14, approved by the in-house Ethics Committee for Animal Experiments, from the University of Zaragoza, Zaragoza, Spain. The care and use of animals were performed accordingly, with the Spanish Policy for Animal Protection RD 53/2013, which meets the European Union Directive 2010/63 on the protection of animals used for experimental and other scientific purposes. Twenty male New Zealand rabbits weighing 2–2.5 kg were kept in a standard rabbit fodder and had free access to water.

### 4.4. Preparation of Ileum Segments and Experimental Protocols

Whole thickness segments (10 mm long) of ileum were removed and vertically suspended in a thermostatically controlled organ bath, containing Krebs solution (in mM: NaCl 120, KCl 4.70, CaCl_2_ 2.40, MgSO_4_ 1.20, NaHCO_3_ 24.50, KH_2_PO_4_ 1.00 and glucose 5.60) at 37 °C to achieve pH 7.4, and continuously gassed with 95% O_2_ and 5% CO_2_. Each segment was connected to an isometric force transducer (Pioden UF1, Graham Bell House, Canterbury, UK). The segments were stretched passively to an initial tension of 2 g. The mechanical activity was amplified (The MacLab Bridge Amp, AD Instruments Inc., Milford, MA, USA), with a range of 2 mV and recorded for further analysis using The MacLab System 8e software (AD Instruments Inc.). The segments were allowed to equilibrate in Krebs solution for 45 min before use. 

After the adaptation period, the spontaneous contractions of longitudinal smooth muscle from ileum were recorded and several experimental protocols were followed: 

To study the effect of EOCM, we added EOCM (1–100 µg·mL^−1^) every 5 min and the cumulative concentration-response curves were performed. 

To compare the effect of EOCM with free Ca^2+^ conditions, papaverine 0.6 mM or free Ca^2+^ Krebs Ringer with EGTA 1 mM was added at the end of some experiments.

To examine the effect of EOCM on contractile agonists, segments of ileum were pre-incubated with EOCM (30 µg·mL^−1^) for 15 min before the addition of carbachol (1 µM) or KCl (60 mM). Some ileum segments were pre-contracted with carbachol (1 µM) or KCl (60 mM) and after 1 min of incubation, EOCM (30 µg·mL^−1^) was added. 

In order to study the role of Ca^2+^ or K^+^ channels on EOCM effect in the spontaneous contractions of ileum, agents such as Bay K8644, trifuoperazine, apamin, tram-34, charybdotoxin, glibenclamide or quinine were added to the bath, 15 min before EOCM (30 µg·mL^−1^). Some segments were also incubated with Bay K8644 for 15 min before the addition of verapamil.

To investigate the involvement of cAMP, cGMP or NO on EOCM responses in the ileum, several agents were added to the bath such as d-cAMP, H-89, forskolin, ODQ(1*H*-[1,2,4]Oxadiazolo[4,3-α]quinoxalin-1-one), cherelythrine, Rp-8-Br-PET-cGMPS, KT-5823, or l-NNA for 15 min before EOCM (30 µg·mL^−1^).

Finally, to examine whether myosin light chain phosphatase, Rho-kinase or hydrogen sulphide participated in EOCM effect on the spontaneous contractions of ileum, some segments were incubated for 15 min with okadaic acid, Y-27632, d,l-propargylglycine or aminooxyacetic acid hemihydrochloride before the addition of EOCM (30 µg·mL^−1^).

### 4.5. Analysis of Data

All intestinal segments included in the analysis showed spontaneous contractions. Thus, each segment served as its own control. The amplitude (in g) and the frequency (contractions per minute, cpm) of spontaneous contractions were measured as previously described [12]. EOCM, ACh, carbachol, KCl or Bay K8644 induced motor responses were measured as the Area Under the Curve (AUC) or as integrated mechanical activity per second, expressed as g s^−1^ and normalized per gram of wet tissue as follows: AUC = AUC_1_ − AUC_0_, where AUC_1_ is the area under the curve per second per gram, either during the first 3 min of response to EOCM, acetylcholine (ACh), carbachol or KCl administration, and AUC_0_ is the area under curve per second per gram of the spontaneous motility, 3 min before adding EOCM or other substances. The AUC values were calculated using a baseline of 0 gram [28].

Data are given as mean percentage with respect to control ± SEM, with n denoting the number of preparations. Comparisons between means were made, using one-way Analysis of Variance (ANOVA) tests, with *p*-values determined using Bonferroni’s post hoc test. The data was analyzed using GraphPad Prism 5.02, and the differences between *p*-values < 0.05 were considered statistically significant.

### 4.6. Chemicals

Acetylcholine (ACh), carbamoylcholine chloride (carbachol), Bay K8644, trifluoperazine dihydrochloride, apamin, charybdotoxin (ChTX), glibenclamide, quinine, verapamil, papaverine dihydrochloride, H-89 dihydrochloride hydrate, KT-5823, chelerythrine chloride, (+)-(*R*)-trans-4-(1-aminoethyl)-*N*-(4-pyridyl) cyclohexanecarboxamide dihydrochloride monohydrate (Y-27632), dibutyryl-cAMP (d-cAMP), d,l-propargylglycine (PAG), aminooxyacetic acid hemihydrochloride (AOAA), tetrodotoxin (TTX) and *N*^G^-nitro-l arginine (l-NNA) were obtained from Sigma (Madrid, Spain). Tram-34, 1*H*-[1,2,4]oxadiazolo [4,3-α]quinoxalin-1-one (ODQ), Rp-8-Br-PET-cGMPS, okadaic acid and forskolin were purchased from Tocris (Madrid, Spain). All chemicals were of analytical grade. Bay K8644 was dissolved in ethanol. Apamin was diluted in acetic acid. Glibenclamide, tram-34, forskolin (Fk), okadaic acid and ODQ were prepared in dimethyl sulfoxide. All other chemicals were dissolved in distilled water.

## Figures and Tables

**Table 1:** Effect of EOCM on the amplitude (%) and frequency (%) of spontaneous contractions of longitudinal smooth muscle of ileum. Results are mean percentage values, with respect to spontaneous contractions in Krebs (control) ± SEM. The number of segments taken from four rabbits is in parentheses.

Conditions	Amplitude %	Frequency %
Control	100 ± 7.5 (14)	100 ± 10.9 (14)
EOCM 1 µg·mL^−1^	84.9 ± 3.4 (14) *	96.7 ± 1.9 (14)
EOCM 3 µg·mL^−1^	72.2 ± 4.2 (14) *	97.3 ± 3.7 (14)
EOCM 10 µg·mL^−1^	62.9 ± 6.1 (14) **	95.5 ± 5.4 (14)
EOCM 30 µg·mL^−1^	40.1 ± 5.4 (14) **	112.3 ± 31 (14)
EOCM 100 µg·mL^−1^	13.1 ± 2.7 (14) **	94.8 ± 41 (14)

* *p* < 0.05; ** *p* < 0.01 *vs.* control.

**Table 2:** Effect of DMSO at several volumes on the area under the curve (AUC, %), amplitude (%) and frequency (%) of spontaneous contractions of longitudinal smooth muscle of the ileum. Results are mean percentage values with respect to spontaneous contractions in Krebs (control) ± SEM. The number of segments taken from four rabbits is in parentheses.

Conditions	AUC (%)	Amplitude (%)	Frequency (%)
Control	100 (15)	100 (15)	100 (15)
DMSO 10 µL	100.8 ± 1.3 (15)	104 ± 2.7 (15)	99.5 ± 1.0 (15)
DMSO 20 µL	100.7 ± 2.7 (15)	104.2 ± 6.3 (15)	98.1 ± 1.9 (15)
DMSO 30 µL	99.0 ± 2.0 (15)	104.1 ± 6.3 (15)	97.7 ± 2.0 (15)
DMSO 40 µL	99.0 ± 2.1 (15)	96.8 ± 4.0 (15)	100 ± 1.3 (15)
DMSO 50 µL	95.2 ± 2.4 (15)	95.4 ± 5.8 (15)	99.2 ± 2.7 (15)
